# Capsaicin Treatment Attenuates Cholangiocarcinoma Carcinogenesis

**DOI:** 10.1371/journal.pone.0095605

**Published:** 2014-04-18

**Authors:** Annika Wutka, Vindhya Palagani, Samarpita Barat, Xi Chen, Mona El Khatib, Julian Götze, Hanane Belahmer, Steffen Zender, Przemyslaw Bozko, Nisar P. Malek, Ruben R. Plentz

**Affiliations:** 1 Department of Internal Medicine I, Medical University Hospital, Tuebingen, Germany; 2 Department of Gastroenterology, Hepatology and Endocrinology, Hannover, Germany; University of Nebraska Medical Center, United States of America

## Abstract

Capsaicin, the most abundant pungent molecule produced by pepper plants, represents an important ingredient in spicy foods consumed throughout the world. Studies have shown that capsaicin can relieve inflammation and has anti-proliferative effects on various human malignancies. Cholangiocarcinoma (CC) is a cancer disease with rising incidence. The prognosis remains dismal with little advance in treatment. The aim of the present study is to explore the anti-tumor activity of capsaicin in cultured human CC cell lines. Capsaicin effectively impaired cell proliferation, migration, invasion, epithelial to mesenchymal transition and growth of softagar colonies. Further, we show that capsaicin treatment of CC cells regulates the Hedgehog signaling pathway. *Conclusion:* Our results provide a basis for capsaicin to improve the prognosis of CCs *in vivo* and present new insights into the effectiveness and mode of action of capsaicin.

## Introduction

Cholangiocarcinoma (CC) is a cancer disease which is increasing worldwide [1], [2]. It represents the second most common primary hepatobiliary cancer and demands a need for a better understanding of the tumor development [3]. Most of the CC tumors are adenocarcinomas arising from epithelial cells lining the intra- and extrahepatic biliary tract system [4], [5]. Known risk factors are primary sclerosing cholangitis (PSC), cirrhosis, chronic viral hepatitis B and C infection, diabetes, obesity, smoking, alcohol intake and toxin exposure like Thorotrast and Dioxins [6]–[8]. CC is usually detected at an advanced stage and patients show up with an extension of the disease which impairs the possibility of curative surgery. Thus, treatment by photodynamic therapy (PDT), systemic chemotherapy and/or radiotherapy are the only options for patients with inoperable disease [9]–[11]. Different studies have shown that CCs are characterized by a series of highly recurrent genetic abnormalities, including KRAS, BRAF, p53, SMAD and p16^INK4a^ mutations [12]–. Currently, the combination of Gemcitabine and Cisplatin is the standard chemotherapeutic regimen for patients undergoing first line treatment [18], [19]. However, standard chemotherapies only offer limited benefit and new strategies are still needed to overcome this deadly disease.

It is well reported that herbal and botanical products, as well as selected food supplements and spices have an anticarcinogenic potential [20]. Capsaicin (*N*-vanillyl-8-methyl-1-nonenamide) is a homovanillic acid derivative and the chief pungent principle found in hot red chilli peppers derived from Capsicum fruit extracts [21]. Capsaicin has an effect on several physiological and pharmacological outcomes [22], [23]. Treatment by capsaicin not only diminishes inflammation and pain, but has also an anti-proliferative effect on different gastrointestinal cancer cells [24]–[35].

However, the exact molecular mechanism for the function of capsaicin in controlling signaling pathways involved in CC carcinogenesis are not described yet. The Hedgehog signaling pathway regulates cell fate decisions, including proliferation, apoptosis, migration and differentiation [36]. Aberrant activation of the Hedgehog pathway has been described for different cancer types [37]–[43]. Studies from our own group and others have recently confirmed the crucial role of Hedgehog in CC carcinogenesis [44]–[48].

In the present study, we sought to further examine the anti-proliferative benefits of capsaicin on different human cholangiocarcinoma cell lines. Our work reveals that capsaicin can block cell proliferation, migration, invasion, colony formation, apoptosis and epithelial-mesenchymal transition (EMT) in human cholangiocarcinoma cells. In addition, we describe for the first time, that capsaicin targets cholangiocarcinoma cells via the Hedghog signaling pathway. Our results suggest that capsaicin may be an effective and promising food supplement with anti-tumor effect against human cholangiocarcinomas.

## Materials and Methods

### Cells Culture

The human cholangiocarcinoma cell lines TFK-1 and SZ-1 were generously provided by Nisar Malek. TFK-1 cells were originally obtained from the DSMZ (German Collection of Microorganisms and Cell Cultures, Human and Animal Cell Lines, Braunschweig, Germany). SZ-1 was established from a surgically resected tumor specimen (from a patient with CC), which was histologically diagnosed as adenocarcinoma of moderate differentiation with cholangiolar differentiation [49]. TFK-1 and SZ-1 cell lines were cultured in RPMI 1640+Glutamax (Invitrogen, Karlsruhe, Germany) supplemented with 10% FCS (Biochrom, Berlin, Germany) and 100 U/ml penicillin/streptomycin (Invitrogen, Karlsruhe, Germany) at 37°C in 5% CO_2_.

### Drugs and Treatment

Capsaicin (Sigma Aldrich, Germany) was prepared as a 100 mM stock in dimethyl sulfoxide, DMSO (AppliChem, Darmstadt, Germany). Cells were treated with DMSO or capsaicin (150 µM, 200 µM).

### Protein extraction and western blotting

SZ1 and TFK1 cells cultured with capsaicin treatment for immunoblots were collected and rinsed with cold phosphate-buffered saline (PBS). Then harvested cells were lysed in lysis buffer containing 20 mM Tris, 150 mM NaCl, 1 mM EDTA, 1 mM EGTA, 1% Triton X-100 and protease and phosphatase inhibitor (Protease Inhibitor Cocktail Tablets, Roche, Mannheim). The concentration of extracted protein was determined using DC protein assay kit (Biorad, München) following manufacturer's instruction. The absorption was measured at 650–750 nm using a microplate reader (Titertek-Berthold, Pforzheim). For immune blotting the cell lysates were loaded at a protein concentration of 30 µl per well. Gel electrophoreses (12% acrylamide gels) was performed (Biorad, München). The membranes were blocked using 5% dried milk (AppliChem, Darmstadt, Germany) for 30 minutes at room temperature. Then they were probed with primary antibodies against E-cadherin (1∶1000; Cell signaling, 24E10), N-cadherin (1∶1000; Millipore), Vimentin (1∶1000; Cell signalling), Smo (1∶1000; Santa Cruz), Gli1 (1∶200; Santa Cruz) and Actin (2∶10.000; Sigma, AC-74), the signal was Detected by AmershamHyperfilm ECL (GE Healthcare Limited, Buckinghamshire, UK). Adobe Photoshop was used to determine the correlative expression of the EMT markers in relation with the loading control Actin. The level of relative expression was further calculated using the data analysis software Microsoft Excel 2002 SP3 (Table S1).

### Proliferation assay

In order to measure the effect of capsaicine on cell proliferation, cells were plated at a concentration of 1×10^4^ cells/ml for in a 96 well plate overnight. Then cells were treated with DMSO and different concentrations of capsaicin (150, 200 µM) for different time points (1–4 days). At the respective time points, 10 µL WST-1 reagents (Roche Diagnostics, Mannheim) was added to each well and incubated for 2 h at 37°C. The absorbance was detected at a wavelength of 492 nm with reference wavelength of 650 nm.

### Invasion assay

Cells (1.25×10^5^ cells/2 ml) were seeded in serum free media into each well of the 6-well BD BioCoat Matrigel Invasion Chamber (BD Biosciences, Bedford, UK). The cells in the inserts were simultaneously treated with capsaicine (150, 200 µM), and the control (DMSO). The inserts were placed into the BD Falcon TC Companion Plate containing 10% FCS and incubated for 48 h hours in a humidified tissue culture incubator, at 37°C, 5% CO_2_ atmosphere. Then the invading cells were fixed with 100% methanol and stained with 1% toluidine blue in 1% borax. Cell were then counted under the microscope (Leica DM 5000 B, Leica, Wetzlar). The calculation of the invading cells were done according to the BD protocol where
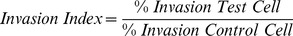



### Migration assay

Cholangiocarcinoma cell lines (SZ-1 and TFK-1) were seeded in a 6-well plate and left to reach 80% confluency. Initially, cells were starved for 24 h in media containing 2% FCS. Then SZ1 and TFK1 were further incubated for 48 h in the starvation media containing either DMSO or capsaicine. Afterwards a scratch was done using a white tip for each treatment. Then cells were washed with PBS and photographed using Leica DMI 6000 B microscope (Leica, Wetzlar). Cells were incubated for an additional 24 h after which the photographs were taken for the wounded area. The migrating cells were calculated according to the following formula:
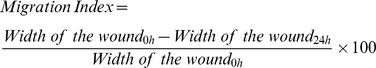



### Anchorage-independent growth assays

Anchorage-independent growth was assayed in SZ-1 and TFK-1 cells to determine the ability of cells to form colonies in soft agar under different treatments. Soft agar plates were prepared in 60 mm plates with bottom layer of 1% nobel agar (Difco; BD Biosciences, Franklin Lakes, NJ, USA) in RPMI 1640+Glutamax (Invitrogen, Karlsruhe, Germany). SZ-1 and TFK-1 cells (6×10^4^ cells/well) were then suspended in medium containing 3 ml of 0.5% agarose along with the respective drug (capsaicin) and control (DMSO) and were seeded as a top layer on to 1% agar coated plates. The cells were incubated for 3 weeks at 37°C in a humidified atmosphere containing 5% CO_2_ and counterstained with p-iodonitotetrazonium violet (Sigma, USA). The number and size of colonies were determined after 3 weeks.

### Detection of Apoptosis by Annexin staining

To determine the apoptosis, cells were seeded (12×10^4^) in six well plate and were further treated under the same conditions described for WST-1 assay. After the respective treatments, floating cells were collected and adherent cells were trypsinized, washed twice with ice-cold PBS. The cells were then resuspended in 1 ml of 1X binding buffer and were stained with Annexin V-FITC and PI according to the manufacture's instruction using Annexin V Apoptosis Detection Kit II (BD Biosciences, San Diego, USA). The signal was detected using LSRFortessa flow cytometer (Becton, Dickinson) and analyzed using FlowJo Version 8.7 software (Tree Star Inc., Ashland, USA).

### Semiquantitative Real-time PCR

RNA was extracted using RNeasy protect Mini Kit (50) (QIAGEN, Hilden). The cDNA was synthesized using iScript cDNA Synthesis Kit (Biorad, USA) from 1 µg total RNa concentration. Semi-quantitative PCR was performed for gli1 (F5′CTCCCGAAGGACAGGTATGTAAC′3/R5′CCCTACTCTTTAGGCACTAGAGTTG′3),gli2(F5′TGGCCGCTTCAGATGACAGATGTTG′3/R5′CGTTAGCCGAATGTCAGCCGTGAAG′3),smo1(F5′GTTCTCCATCAAGAGCAACCAC′3/R5′CGATTCTTGATCTCACAGTCAGG′3) and 18-sRNA(F5′AAACGGCTACCACATCCAAG′3/R5′ CCTCCAATGGATCCTCGTTA′3) with product sizes of 248, 200, 258, and 155 respectively. The PCR was carried at an annealing temperature of 55°C for 32 cycles. The Adobe Photoshop was used to determine the correlative expression of the Hedgehog targets in relation with the loading control GAPDH. The level of relative expression was further calculated by using the data analysis software Microsoft Excel 2002 SP3 (Table S2).

### Isolation and immortalization of human liver Fibroblasts by lentiviral mediated gene transfer

HEK- 293 T cells were transfected by Calcium phosphate method with two packaging plasmids pCMV-VSV-G (Addgene Plasmid 8454, Cambridge, UK), pCMV-dR8.2 dvpr (Addgene Plasmid 8455, Cambridge, UK) and a target vector pLenti CMV/TO SV40 small+Large T (w612-1) (Addgene Plasmid 22298, Cambridge, UK) [50]. Viruses were harvested after 48 h and 72 h followed by infection of primary Fibroblasts. Efficiency of SV40 L T – Antigen gene transfer was determined by semiquantitative PCR. The expression of fibroblast specific markers (N-Cadherin, Fibronectin, CD90) were evaluated by quantitative realtime PCR. Human fibroblasts were cultured under same conditions like TFK-1 and SZ-1 cells.

### Ethics Statement

Use of human tissue was reviewed by local ethic committee of Medical School of Hannover (MHH) (protocol no: SZ 11/203) and written consent form from the donor was obtained. MHH approved the usage of human tissue for the underlying study.

### Statistical Analysis

Experiments were repeated 3 times. The results were analyzed using software Graphpad prism version 5.0 (GraphPad Software, San Diego, CA, USA) and SPSS Version 11.0 (SPSS, Chicago, USA). The tests include one way ANNOVA analysis of variance and student's *t*-test along with Bonferroni post test and paired and unpaired t-tests. Differences were considered as statistically significant when the P-value was less <0.05 (*), <0.005 (**) and <0.001 (***).

## Results

### Capsaicin decreases the viability of human cholangiocarcinoma cell lines and induces apoptosis

**Figure 1:**
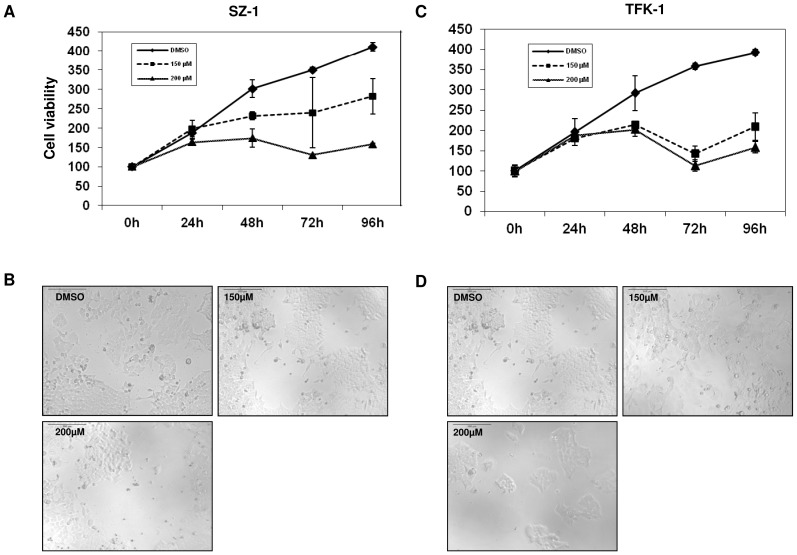
Capsaicin inhibits cell proliferation in human cholangiocarcinoma cell lines. The cell proliferation of (A) SZ1 and (C) TFK-1 cells was measured by cell proliferation assay. Capsaicin (150 µM, 200 µM) inhibited cell proliferation in a dose- and time-dependent manner. Light microscopic pictures (10× magnification) were taken at 96 h to show the effect of capsaicin on cell proliferation of (B) SZ1 and (D) TFK-1. Note that these results reveal the anti-proliferative effects of capsaicin on human cholangiocarcinoma cells. Data are expressed as mean ± SD of triplicates.

**Figure 2:**
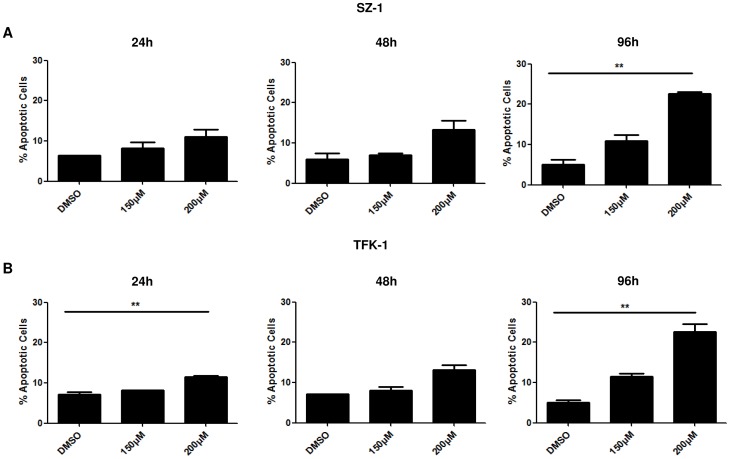
Capsaicin induces apoptosis of cholangiocarcinoma cells. SZ-1 (A) and TFK-1 (B) cells were treated with capsaicin or DMSO as control for 24 h, 48 and 96 h. Viable cells were collected and were stained by Annexin V/PI dye. The stained cells were determined by flow cytometry. Data are representing the results of two independent experiments. Data are expressed as mean ± SD of triplicates.

To test the pharmacological potential of capsaicin on the proliferation of cholangiocarcinomas, two human cell lines (SZ-1 and TFK-1) were analyzed by WST assay. We determined the IC50 by constructing a dose-response growth curve and examined the effect of different concentrations of capsaicin on CC cells (Figure S1 A,B). Taking into account the calculated IC50 and other publications in this field we have used capsaicin dosages between 150 and 200 µM [31], [32], [51], [52]. As illustrated in Figure 1, capsaicin treatment for 24, 48, 72 and 96 h reduced the number of viable SZ-1 and TFK-1 cells in a dose- and time- dependent manner (Figure 1 A–D). Phase contrast images confirms the cell proliferation results (Figure 1 B,D). In order to investigate whether the anti-tumor effects of capsaicin are specific only to the malignant cells or this drug also affects the normal cells, we performed an IC50 experiment using human fibroblasts as a normal cell line. The calculated IC50 of the malignant lines (SZ-1 and TFK-1) were found to be different from normal cells (human fibroblast). Moreover, capsaicin treatment affected cell viability of human fibroblasts only in higher concentrations (Figure S1 C). To elucidate whether the decrease observed in the proliferation and viability of cholangiocarcinoma cells after capsaicin treatment was associated with induction of apoptosis, annexin staining was performed and the percentage of apoptotic cells was assessed by FACS analysis. The results show that treatment by different concentrations of capsaicin for the indicated time points induces apoptosis both in SZ-1 (Figure 2 A) and TFK-1 (Figure 2 B) cells. Importantly, we observed a significant difference in apoptotic cells after 96 h in SZ-1 and 24+96 h in TFK-1 cells. Our data suggest that capsaicin inhibits the growth in dose- and time-dependent manner and that apoptosis could be the reason for the cells that makes the proliferation number goes down. However, in our study we have focused only on the programmed cell death. The exact way of cell death can be of any type which definitely requires further studies.

### Treatment by capsaicin results in the inhibition of human cholangiocarcinoma cell migration, invasion and colony formation

**Figure 3:**
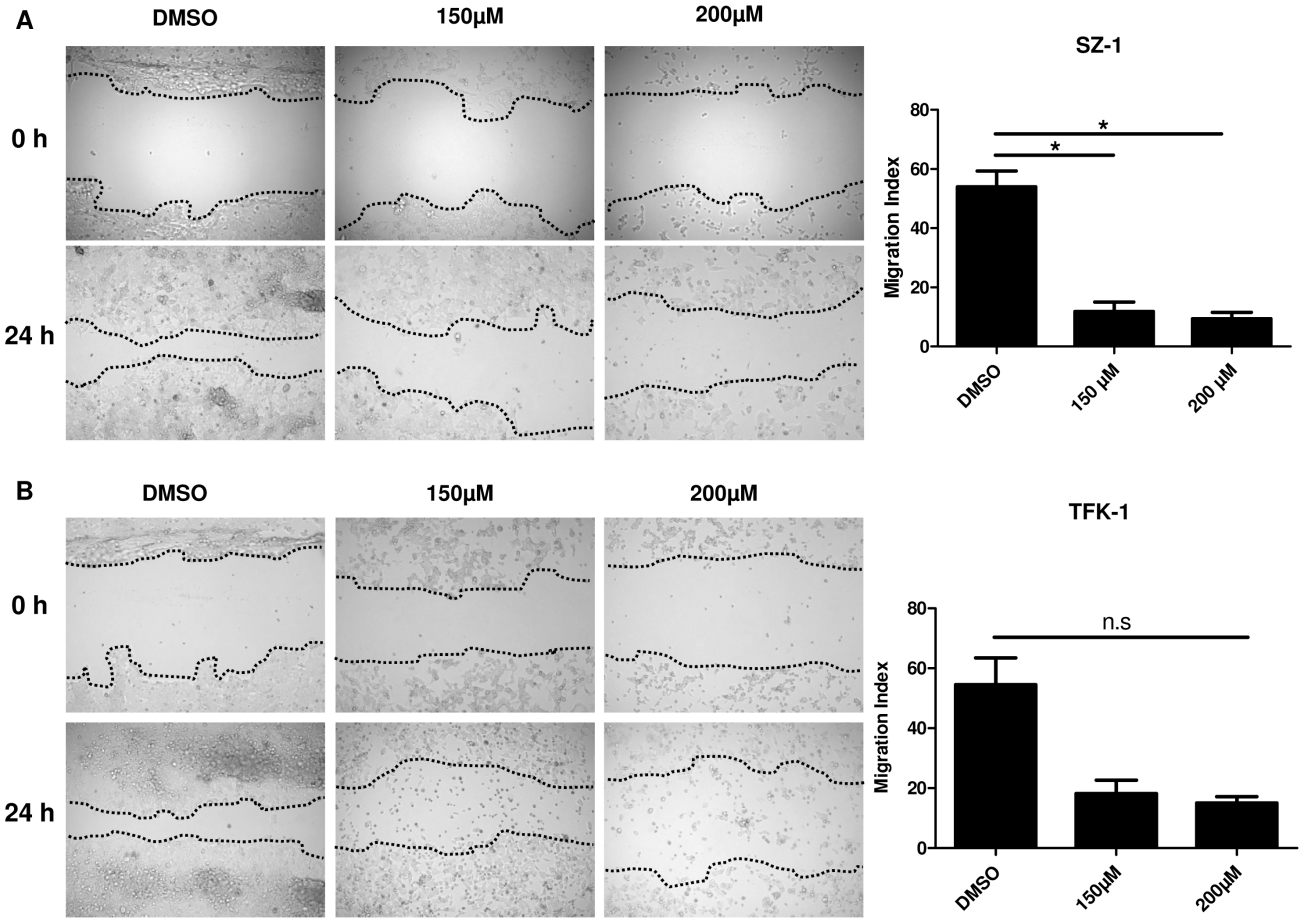
Capsaicin attenuates migration of human cholangiocarcinoma cells. Treatment with capsaicin suppresses the migration potential of human cholangiocarcinoma cell lines SZ-1 and TFK-1. Wound healing experiments of (A) SZ1 and (B) TFK cells cultured with capsaicin (150 µM, 200 µM) or control (DMSO). A scratch was made at (time 0 h) in both SZ-1 and TFK-1 and maintained for 24 h in conditioned medium with capsaicin or DMSO. The dotted lines are representing the edges of the wound. Photographs were taken under light microscope (10× magnification). After 24 h (A) SZ1 and (B) TFK-1 showed significant inhibition under 150 µM and 200 µM capsaicin. In DMSO treated cells 90% of the wound healing was observed after 24 hrs. (A,B) The migration index was calculated as described in Material and Methods and plotted in bar graphs. P values were calculated with ANOVA analysis of variance along with Bonferroni post test. The error bar represents standard deviation. Differences were considered as statistically significant (*) when the P-value was less <0.05. Data are expressed as mean ± SD of triplicates.

**Figure 4:**
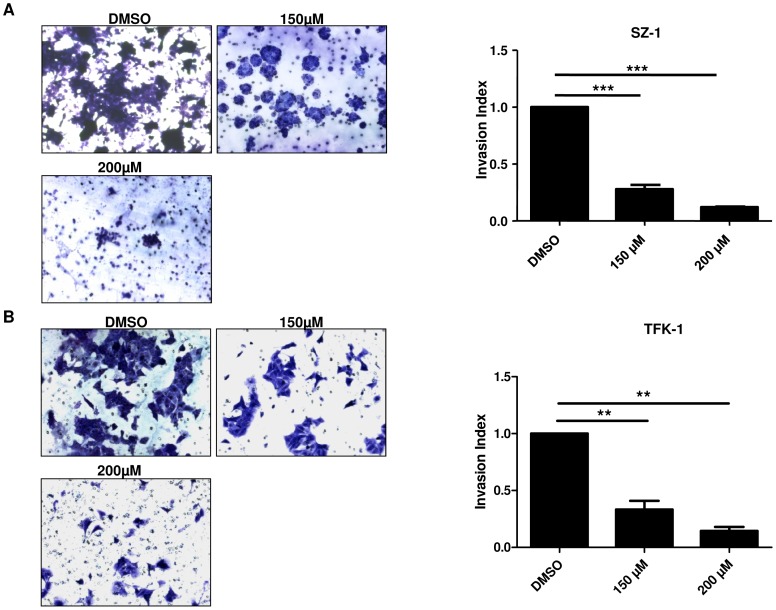
Invasion activity of human cholangiocarcinoma cells in response to capsaicin treatment. SZ-1 (A) and TFK-1 (A) cell lines were treated for 48 h with control (DMSO) and capsaicin (150 µM, 200 µM) to investigate the effect of capsaicin on invasiveness of human cholangiocarcinoma cells. The number of cells that invaded through the membrane was determined by light microscope (20× magnification) counterstained and invasion index (A,B) was calculated as described in Material and Methods and plotted in bar graphs. Both TFK-1 and SZ-1 showed significant decrease in number of invading cells by light microscope. P values were calculated with ANOVA analysis of variance along with Bonferroni post test. The error bar represents standard deviation. Differences were considered as statistically significant when the P-value was less <0.005 (**) and <0.001 (***). Data are expressed as mean ± SD of triplicates.

**Figure 5:**
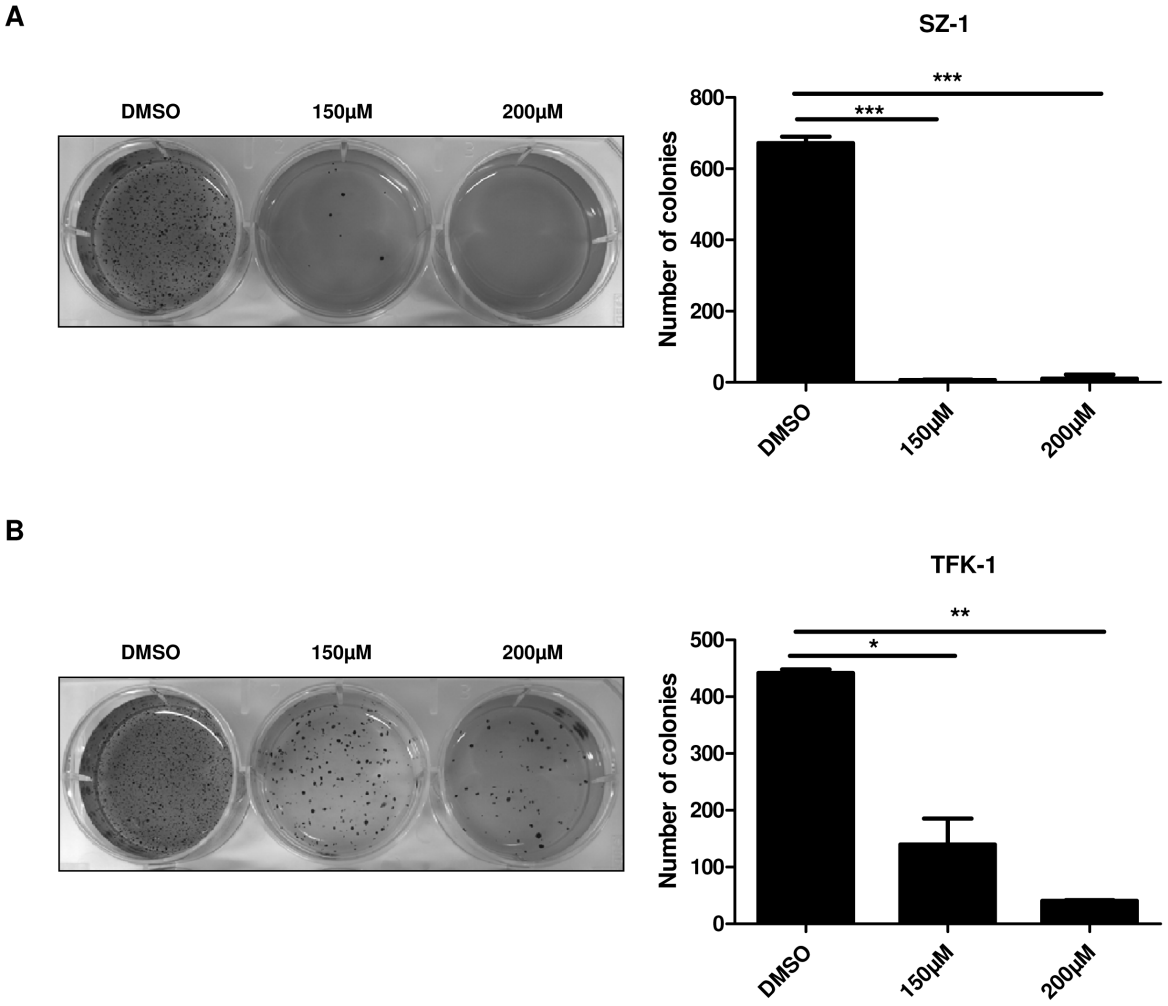
Capsaicin treatment suppresses the colony formation ability of cholangiocarcinoma cells. Soft agar assay was performed in capsaicin-treated human cholangiocarcinoma cells SZ-1 (A) and TFK-1 (B), with quantification. Compared to DMSO (control) capsaicin inhibits colony formation at a concentration starting at 150 µM. P values were calculated with ANOVA analysis of variance along with Bonferroni post test. The error bar represents standard deviation. Differences were considered as statistically significant when the P-value was less <0.05 (*), <0.005 (**) and <0.001 (***). Data are expressed as mean ± SD of triplicates.

We next examined the effect of capsaicin (150 µM, 200 µM) on cell motility using *in vitro* wound healing assays. Cells were treated with similar concentrations and combinations of drugs as mentioned above. Significant (p<0.05) inhibition of wound healing was observed with 150 µM and 200 µM capsaicin in SZ-1 cells (Figure 3 A). Migration of TFK-1 cells showed a strong tendency to be impaired by capsaicin, but the results were not significant (Figure 3 B). In contrast, ca. 80% wound healing was seen after 24 h in DMSO cells. Furthermore, we performed cell invasion using Matrigel-coated transwell chambers under DMSO and capsaicin treatments (150 µM and 200 µM) and experiments were conducted as described in Material and Methods. As shown in Figure 4 A-B, capsaicin inhibited significantly cell invasion in a dose dependent manner. Around 90% decrease in the number of invading cells was observed compared to the control group. Finally, we examined the effect of capsaicin treatment on anchorage independent growth by assaying colony formation on of SZ-1 and TFK-1 cells on soft agar (Figure 5 A, B). The results have shown that cells were inhibited from forming colonies under different dose of capsaicin compared to DMSO. These results suggest that capsaicin prevents migration, invasion and colony formation of human CC cells.

### Capsaicin impairs epithelial mesenchymal transition in human cholangiocarcinoma cell lines

**Figure 6:**
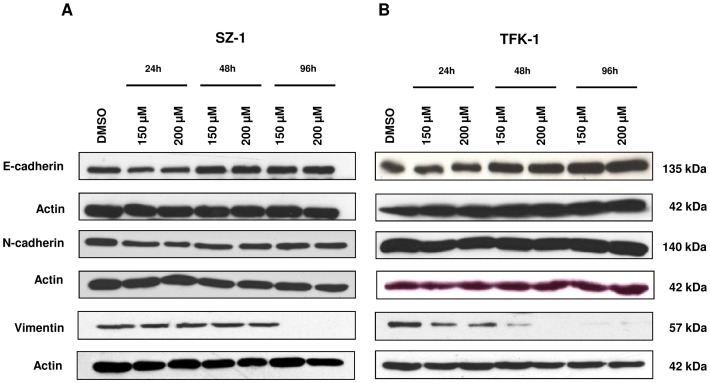
Capsaicin impairs epithelial mesenchymal transition. (A) SZ-1 and (B) TFK-1 cells were treated with control (DMSO) and capsaicin (150 µM, 200 µM) for 24 h, 48 h and 96 h. The expression of EMT markers: E-cadherin, N-cadherin and Vimentin were analyzed by Western blot. β-actin was used as a loading control. (A) SZ1 and (B) TFK1 cells showed an increase of epithelial marker E-cadherin and a dose- and time-dependent decrease of Vimentin. N-cadherin expression was nearly unchanged for both cholangiocarcinoma cell lines.

In order to further examine whether capsaicin has an effect on EMT in human CC cell lines, SZ-1 and TFK-1 cells were treated with different capsaicin concentrations (150 µM, 250 µM) for the indicated time points and the expression of EMT markers were evaluated by western blot. The quantification of the western blot expression is shown in Table S1. Capsaicin treatment resulted in a time-dependent increase of the epithelial marker E-cadherin especially for TFK-1 cells and a dose- and time-dependent decrease of Vimentin for both cell lines as assessed by Western Blot (Fig. 6 A, B, Table S1). However, there was only a change in the expression of the mesenchymal marker, N-cadherin in SZ-1 cell at 24 h (Fig. 6 A, B, Table S1). Therefore, these data show that capsaicin treatment could modulate partially EMT phenotype in human cholangiocarcinoma cell lines.

### Capsaicin therapy targets Hedgehog signaling

**Figure 7:**
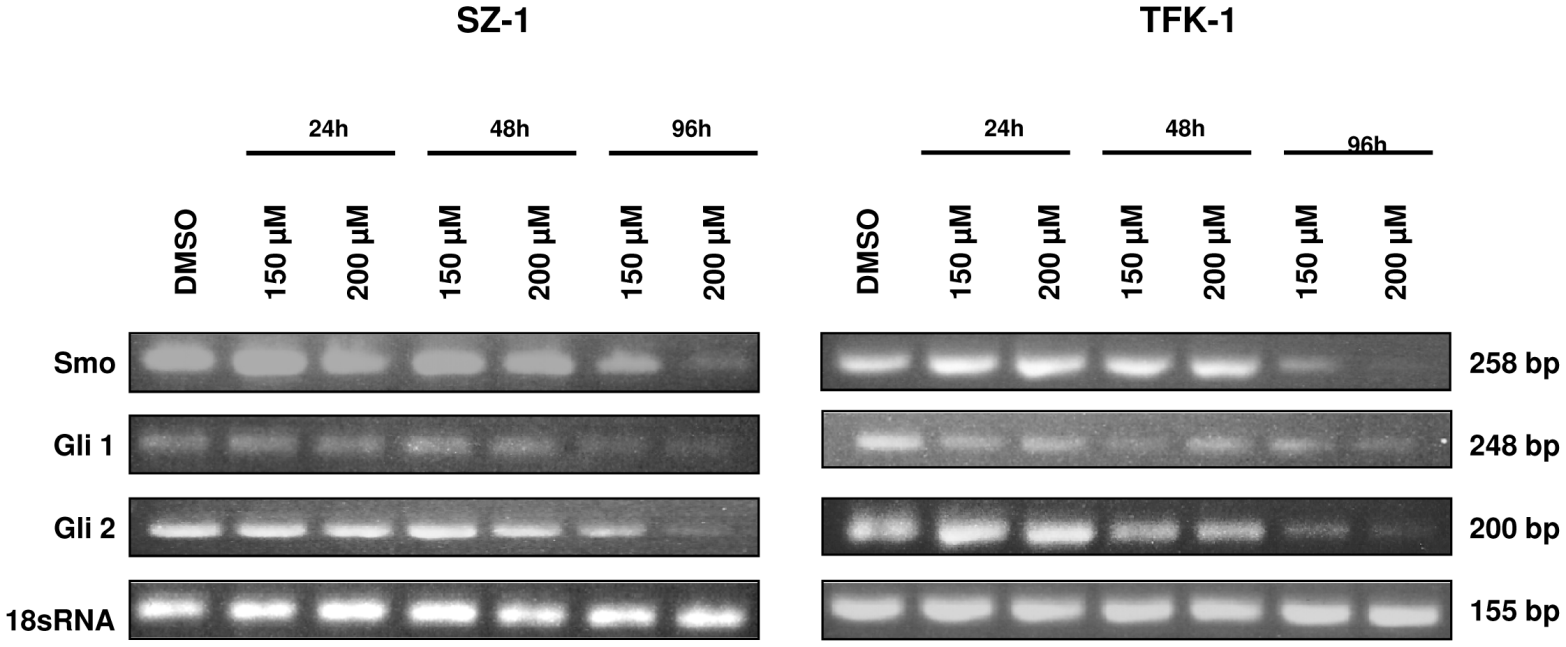
Capsaicin targets Hedgehog signaling. The expression levels of Smo, Gli1 and Gli2 were analyzed by semiquantitative RT-PCR both in SZ-1 (A) and in TFK-1 cells (B) after treatment either with control (DMSO) or capsaicin (150 µM, 200 µM) for 24 h, 48 h and 96 h. A reduction of transmembrane protein Smo was seen in both cell lines after 96 h. Capsaicin down-regulates Hedgehog targets Gli1 and Gli2 in a time-dependent manner (96 h).

The anti-proliferative effect of capsaicin on cholangiocarcinoma cells is not fully identified yet. Hedgehog signalling has been implicated in the invasive growth of human cholangiocarcinoma cells. To gain more insights into its effects, we determined the expression of the targets of the Hedgehog signaling pathway. In both cell lines, capsaicin treatment was correlated with a time-dependent down-regulation of the mRNA expression of the Hedgehog targets Gli1 and Gli2, especially for TFK-1 cells (Fig. 7 A, B). By semiquantitative real-time-PCR we could also detect a change of the RNA expression of the transmembrane protein Smo after 96 h. The quantification of the real-time-PCR is shown in Table S2. The expression on protein level was also analyzed for Gli1 and Smo and the results confirmed the same tendency like on RNA level (Figure S2, Table S1). These results suggest that capsaicin interferes with the growth and proliferation and viability of human cholangiocarcinoma through targeting the Hedgehog signalling pathway. However, the results were different between both CC cell lines.

## Discussion

Several studies have shown a chemopreventive potential of capsaicin, a plant phytochemical in chilli peppers [24]–[35]. Diverse mechanisms have been postulated for the anti-tumor effects of capsaicin in gastro-intestinal cancer [53]. However, the relationship of capsaicin to the carcinogenicity in human CCs has not been analyzed yet. In the present study, we evaluated the chemopreventive potential of capsaicin against CC. We provide first evidence that capsaicin efficiently inhibited the growth in human intrahepatic (SZ-1) and extrahepatic (TFK-1) cholangiocarcinoma cells. Capsaicin therapy also induces apoptosis and attenuates the Hedgehog signaling pathway in CC.

It has been previously demonstrated that capsaicin induced apoptosis in Hep-1, HepG2 and pancreatic cancer cells through caspase-3 dependent mechanism [29], [31], [32]. It was also reported that capsaicin modulates cell cycle progression and apoptosis in human KB cancer and bladder carcinoma cells [51], [52]. In agreement with these data we found that capsaicin also induced apoptotic cell death in CC cells. However, treatment did not cause high apoptosis rates and is an indication of the capacity of other restrictions of cell death, which requires further studies. Epithelial-to-mesenchymal transition (EMT) is the collection of events that allows the conversion of adherent epithelial cells into independent fibroblastic cells possessing migratory properties and the ability to invade the extracellular matrix [54]. E-cadherin, a protein modulated during EMT, is known to be expressed by liver progenitors and biliary epithelial cells [55]. In extrahepatic cholangiocarcinoma, Araki et al. showed in extrahepatic cholangiocarcinoma that the cadherin switch promotes tumor progression via TGF-β signaling [56]. In contrast to Yang et al, we demonstrated that capsaicin partially influenced EMT [57]. Our results showed that E-cadherin is essential for the capsaicin mediated inhibition of invasion and migration. Capsaicin treatment resulted also in dose-dependent decreased expression of Vimentin a marker of mesenchymally-derived cells. Interestingly, we could not detect any down regulation of the mesenchymal marker N-cadherin, arguing for a selective control of EMT by capsaicin. Additionally, capsaicin treated cholangiocarcinoma cells had a great reduction in the capacity to form colonies.

Since it is known that Hedgehog signaling is deregulated in many cancers, including cholangiocarcinomas, we tested whether capsaicin might target also the Hedgehog pathway. Hedgehog is a major regulator for cell differentiation, tissue polarity and cell proliferation in embryonic development and homeostasis in adult tissues [36]–[44]. It is reported that targeted inhibition of Hedgehog signaling is effective against cancer growth [36]–[44]. Hedgehog inhibitors mainly target the Hedgehog pathway by neutralizing the activity of Hedgehog ligands, inhibiting the serpentine receptor Smoothened (SMO), and inhibiting the activity of Gli transcription factors [36]–[44]. Bai et al. reported that in a mouse model of pancreatic intraepithelial neoplasia, capsaicin treatment results in a decrease of Shh and Gli1 mRNA via inhibiting pancreatitis [58]. In our study, capsaicin also targets the Hedgehog signaling pathway on RNA and protein level. In both studied CC cell lines, capsaicin treatment was correlated with a down-regulation of the Hedgehog targets Gli1 and Gli2. Also the expression of SMO was affected in a time-dependent manner. However, with our data we could not decipher if capsaicin impairs the activation of the autocrine or paracrine Hedgehog signaling. We also could not rule out that capsaicin has some other indirect anti-tumor effects.

Taken together, our results indicate that capsaicin demonstrates strong activities against cell proliferation and *in vitro* carcinogenesis by blocking Hedgehog pathway activation. The use of capsaicin as a food supplement to inhibit Hedgehog signaling might therefore be of additional therapeutic benefit in patients with CC.

## Supporting Information

Figure S1 **IC50 concentrations for SZ-1, TFK-1 and human fibroblasts.** Viable cells were quantified using WST assay and the concentrations exiting 50% inhibition (IC50) were calculated. (A) IC50 calculation for SZ-1. (B) IC50 calculation for TFK-1. (C) IC50 calculation for human fibroblasts and corresponding light microscope pictures (magnification 10×) at 96 hours.
(TIF)

Figure S2 **Capsaicin targets Hedgehog signaling on protein level.** (A) SZ-1 and (B) TFK-1 cells were treated with control (DMSO) and capsaicin (150 µM, 200 µM) for 24 h, 48 h and 96 h. The expression of Hedgehog targets: Smo and Gli1 were analyzed by Western blot. β-actin was used as a loading control. (A) SZ1 showed a decrease of Smo at 96 hours and of Gli1 at 24 hours. (B) TFK-1 showed a decrease of Smo at 24 hours and of Gli1 at 96 hours.
(TIF)

Table S1 **Quantification of protein expression results.**
(DOC)

Table S2 **Quantification of semiquantitative RT-PCR results.**
(DOC)
