# Correction: Capsaicin Treatment Attenuates Cholangiocarcinoma Carcinogenesis

**DOI:** 10.1371/journal.pone.0162673

**Published:** 2016-09-20

**Authors:** Annika Wutka, Vindhya Palagani, Samarpita Barat, Xi Chen, Mona El Khatib, Julian Götze, Hanane Belahmer, Steffen Zender, Przemyslaw Bozko, Nisar P. Malek, Ruben R. Plentz

There is an error in Fig 1 of the published article. The DMSO image in Fig 1D is a duplicate of the 150μM image in Fig 1B and was incorrectly included in Fig 1D. Please see the correct Fig 1 here.

**Fig 1:**
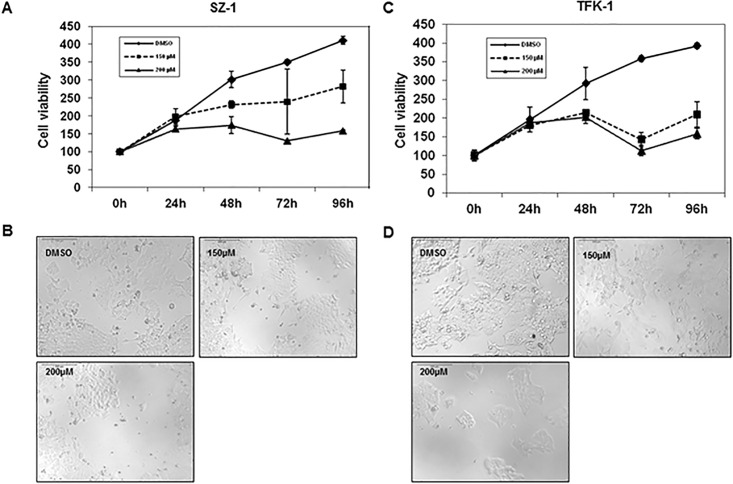
Capsaicin inhibits cell proliferation in human cholangiocarcinoma cell lines. The cell proliferation of (A) SZ1 and (C) TFK-1 cells was measured by cell proliferation assay. Capsaicin (150 μM, 200 μM) inhibited cell proliferation in a dose- and time-dependent manner. Light microscopic pictures (10× magnification) were taken at 96 h to show the effect of capsaicin on cell proliferation of (B) SZ1 and (D) TFK-1. Note that these results reveal the anti-proliferative effects of capsaicin on human cholangiocarcinoma cells. Data are expressed as mean ± SD of triplicates.
