# Supplementary figures and images for: Capsaicin Treatment Attenuates Cholangiocarcinoma Carcinogenesis
Source: PLoS One. 2014 Apr 18;9(4):e95605. doi: 10.1371/journal.pone.0095605 (PMC3991659; doi:10.1371/journal.pone.0095605)

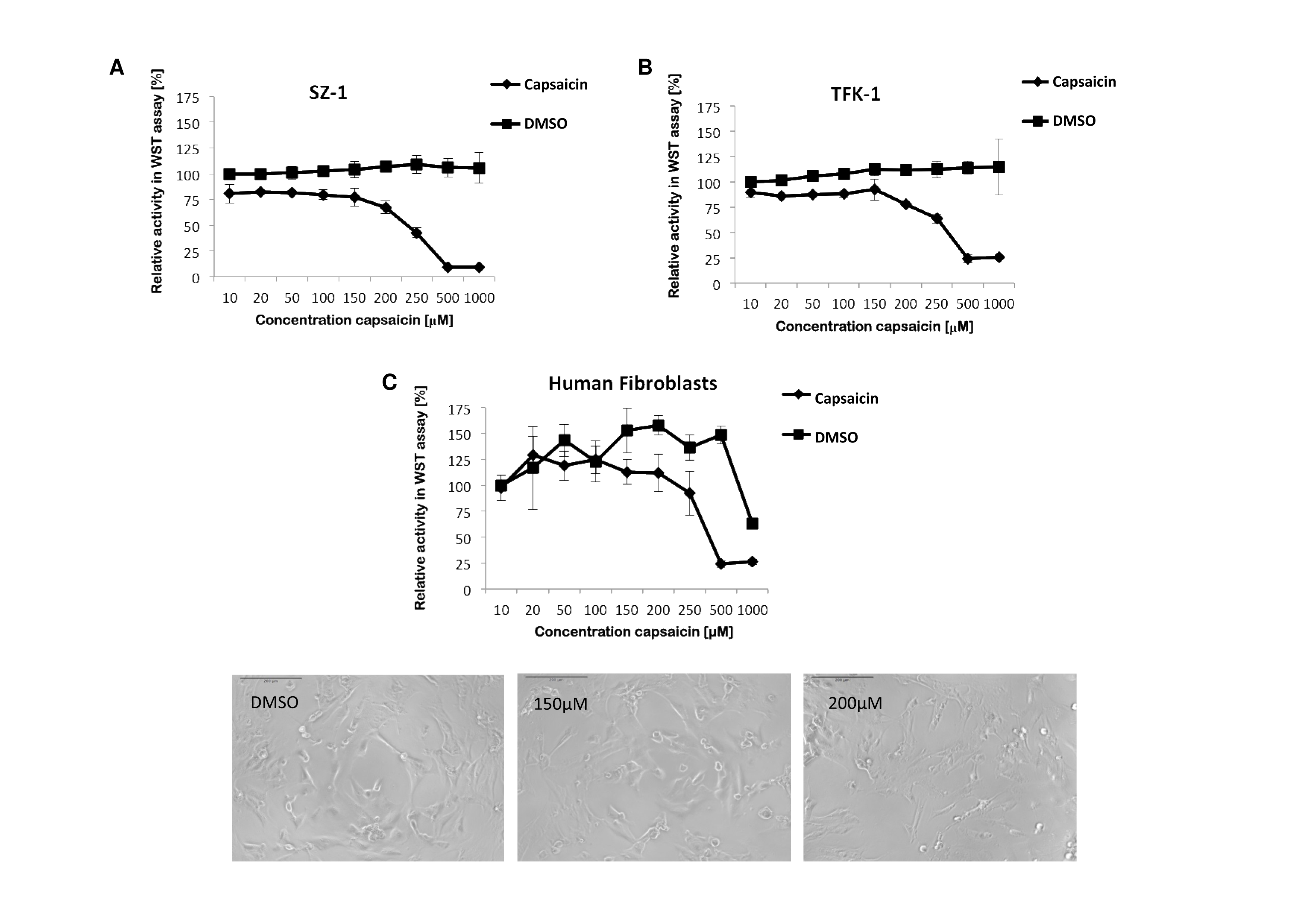

Supplement: Figure S1 — IC50 concentrations for SZ-1, TFK-1 and human fibroblasts. Viable cells were quantified using WST assay and the concentrations exiting 50% inhibition (IC50) were calculated. (A) IC50 calculation for SZ-1. (B) IC50 calculation for TFK-1. (C) IC50 calculation for human fibroblasts and corresponding light microscope pictures (magnification 10×) at 96 hours. (TIF) [file pone.0095605.s001.tif]

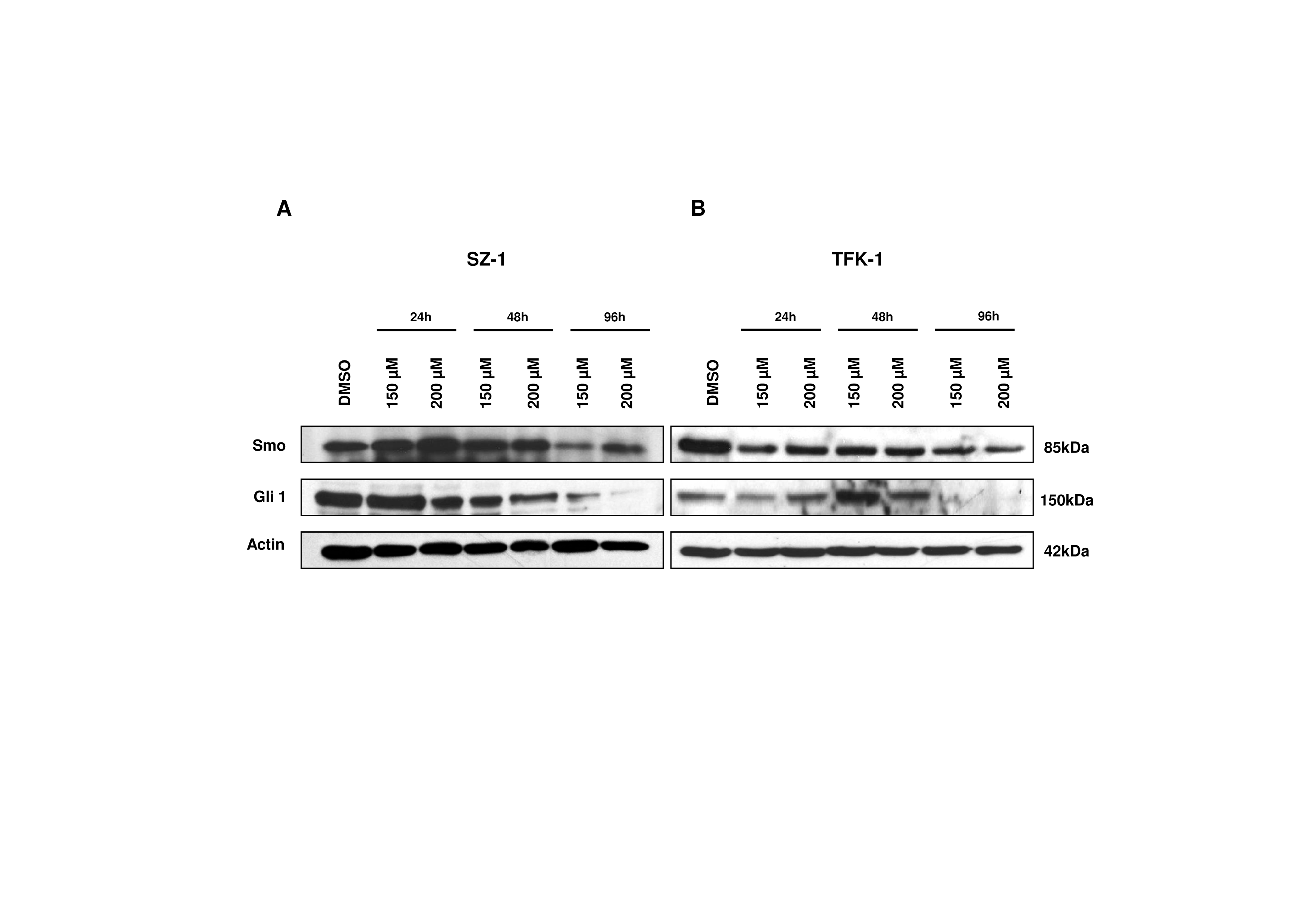

Supplement: Figure S2 — Capsaicin targets Hedgehog signaling on protein level. (A) SZ-1 and (B) TFK-1 cells were treated with control (DMSO) and capsaicin (150 µM, 200 µM) for 24 h, 48 h and 96 h. The expression of Hedgehog targets: Smo and Gli1 were analyzed by Western blot. β-actin was used as a loading control. (A) SZ1 showed a decrease of Smo at 96 hours and of Gli1 at 24 hours. (B) TFK-1 showed a decrease of Smo at 24 hours and of Gli1 at 96 hours. (TIF) [file pone.0095605.s002.tif]
