# Supplementary material for: Capsaicin Treatment Attenuates Cholangiocarcinoma Carcinogenesis
Source: PLoS One. 2014 Apr 18;9(4):e95605. doi: 10.1371/journal.pone.0095605 (PMC3991659; doi:10.1371/journal.pone.0095605)
Supplement: Table S1 — Quantification of protein expression results. (DOC) [file pone.0095605.s003.doc]

**Table S1. Quantification of protein expression results.**

| ***SZ-1*** | **DMSO** | **150µM (24h)** | **200µM (24h)** | **150µM (48h)** | **200µM (48h)** | **150µM (96h)** | **200µM (96h)** | Relative intensity |
| --- | --- | --- | --- | --- | --- | --- | --- | --- |
| E-cadherin | 100 | 76.55 | 86.29 | 110.77 | 110.48 | 105.38 | 118.01 | (%) |
| N-cadherin | 100 | 86.89 | 69.95 | 99.70 | 97.09 | 99.65 | 106.03 | (%) |
| Vimentin | 100 | 92.55 | 97.77 | 108.82 | 104.25 | 0 | 0 | (%) |
| Gli1 | 100 | 105.43 | 78.28 | 77.03 | 76.53 | 31.61 | 11.40 | (%) |
| Smo | 100 | 113.85 | 115.50 | 120.34 | 125.11 | 75.20 | 117.52 | (%) |
| ***TFK-1*** |  |  |  |  |  |  |  |  |
| E-cadherin | 100 | 95.04 | 100.34 | 127.42 | 129.69 | 145.70 | 155.03 |  |
| N-cadherin | 100 | 96.85 | 94.21 | 94.49 | 91.21 | 96.87 | 90.64 | (%) |
| Vimentin | 100 | 44.18 | 44.60 | 22.24 | 0 | 14.57 | 16.03 | (%) |
| Gli1 | 100 | 63.27 | 90.12 | 143.68 | 135.17 | 0 | 0 | (%) |
| Smo | 100 | 41.33 | 47.50 | 46.97 | 53.58 | 43.60 | 29.92 | (%) |
