# Supplementary material for: Capsaicin Treatment Attenuates Cholangiocarcinoma Carcinogenesis
Source: PLoS One. 2014 Apr 18;9(4):e95605. doi: 10.1371/journal.pone.0095605 (PMC3991659; doi:10.1371/journal.pone.0095605)
Supplement: Table S2 — Quantification of semiquantitative RT-PCR results. (DOC) [file pone.0095605.s004.doc]

| ***SZ-1*** | **DMSO** | **150µM (24h)** | **200µM (24h)** | **150µM (48h)** | **200µM (48h)** | **150µM (96h)** | **200µM (96h)** | **Relative intensity** |
| --- | --- | --- | --- | --- | --- | --- | --- | --- |
| Gli1 | 100 | 146.99 | 122.75 | 163.59 | 170.13 | 65.48 | 82.58 | (%) |
| Gli2 | 100 | 89.11 | 79.14 | 83.15 | 54.30 | 37.51 | 17.35 | (%) |
| Smo | 100 | 99.05 | 91.56 | 91.27 | 96.71 | 88.65 | 67.28 | (%) |
| ***TFK-1*** |  |  |  |  |  |  |  |  |
| Gli1 | 100 | 80.24 | 82.23 | 77.50 | 92.48 | 85.56 | 70.47 | (%) |
| Gli2 | 100 | 93.35 | 70.64 | 48.94 | 34.88 | 10.81 | 10.73 | (%) |
| Smo | 100 | 115.63 | 117.70 | 105.63 | 108.27 | 53.25 | 32.79 | (%) |

**Table S2. Quantification of semiquantitative RT-PCR results.**
